# Glycerol phenylbutyrate as an effective chemical chaperone for traffic-defective ABCB4 variants: comparative analysis with 4-phenylbutyrate

**DOI:** 10.1186/s13023-026-04663-1

**Published:** 2026-11-10

**Authors:** Manon Banet, Yosra Riahi, Emmanuel Gonzales, Emmanuel Jacquemin, Martine Lapalus, Thomas Falguières

**Affiliations:** 1 Université Paris-Saclay, Inserm, Physiopathogénèse et traitement des maladies du foie, UMR_S 1193, Bâtiment Henri Moissan – 17, avenue des Sciences, Orsay, F-91400 France; 2 Université Paris-Saclay, Faculté de Médecine, AP-HP, Bicêtre Hospital, Paediatric Hepatology & Paediatric Liver Transplant Department, Reference Center for Rare Paediatric Liver Diseases, FILFOIE, ERN RARE LIVER, Le Kremlin-Bicêtre, F- 94270 France

**Keywords:** ABC transporters, Bile secretion, Cholestatic liver diseases, Targeted pharmacotherapy, MDR3, Progressive familial intrahepatic cholestasis

## Abstract

The ABCB4 transporter is localised at the canalicular membrane of hepatocytes, where it mediates the secretion of phosphatidylcholine into bile. Genetic variations in the *ABCB4* gene are responsible for rare cholestatic liver diseases of varying severity, the most severe form being progressive familial intrahepatic cholestasis type 3. Existing treatments do not cure most patients with this disease, who require liver transplantation before adulthood. Consequently, there is a need to develop alternative pharmacological therapies. 4-phenylbutyrate and glycerol phenylbutyrate can partially correct the mistrafficking of endoplasmic reticulum-retained ABCB11 variants, thereby improving the clinical condition of patients with ABCB11 deficiency, but in vitro and in vivo data on ABCB4 are limited. In this study, we tested the ability of these two chemical chaperones to rescue traffic-defective ABCB4 variants in vitro by assessing their effects on subcellular localisation and phospholipid efflux capability, which revealed a greater rescuing capacity of glycerol phenylbutyrate over 4-phenylbutyrate. However, the non-equimolar doses used here preclude a direct comparison of the relative efficacy of the two molecules.

## Introduction

Bile secretion is a central physiological function of the liver, essential for the emulsification and absorption of dietary lipids and the elimination of xenobiotics and endogenous metabolites [1]. This process relies on the coordinated action of membrane transporters of the large family of ATP-binding cassette (ABC) transporters, which use ATP hydrolysis to actively translocate their substrates across the canalicular membrane of hepatocytes. Among these transporters, the ATP-binding cassette subfamily B member 4 (ABCB4) - also known as multidrug resistance type 3, or MDR3 - specifically ensures the flopping of phosphatidylcholine (PC) from the inner leaflet to the outer leaflet of the canalicular membrane, allowing PC to be incorporated into bile by forming mixed micelles with bile salts and cholesterol, co-secreted by ABCB11 and ABCG5/G8, respectively [2]. Genetic variations in the *ABCB4* locus lead to ABCB4 deficiency and the secretion of a lithogenic and PC-poor bile, thereby promoting the formation of cholesterol crystals and gallstones in the biliary tract, and a loss of protection against the detergent effects of free bile salts on biological membranes of the biliary tree, which leads to several pathologies of varying severities [3], the most severe and rare form of which being progressive familial intrahepatic cholestasis type 3 (PFIC3). PFIC3 is a rare autosomal recessive disease that manifests within the first months or years of life and is frequently associated with progressive liver injury, ultimately leading to cirrhosis and end-stage liver disease [3]. Current first-line therapy relies on ursodeoxycholic acid (UDCA), a relatively hydrophilic bile acid, which can alleviate cholestasis and improve clinical outcomes in patients with milder forms of the disease [4]. However, UDCA shows limited or no efficacy in severe forms of the disease, in which progression to liver failure is common, and liver transplantation often represents the only curative option [5]. These limitations underscore the urgent need to develop novel pharmacological strategies. In the context of drug repurposing, sodium 4-phenylbutyrate (4PB), a molecule approved by both the European Medicines Agency and the Food and Drug Administration and currently used as an ammonia scavenger (Ammonaps™, Pheburane™) in patients with urea cycle disorders (UCD), has been used. 4PB has been shown to increase cell surface expression of the F508del variant of the cystic fibrosis transmembrane conductance regulator (CFTR/ABCC7) [6, 7], as well as the canalicular expression of missense variants and wild type (WT) form of ABCB11 in vitro, thus improving the condition of patients with ABCB11 deficiency – PFIC2 [8, 9]. These data provided evidence that 4PB can act as a chemical chaperone able to correct plasma membrane targeting of endoplasmic reticulum (ER)-retained proteins.

However, the long-term use of 4PB is frequently limited by poor tolerability, as its unpleasant palatability is associated with significant emetic effects, while its formulation requires the ingestion of a large number of pills (up to 40 per day) thereby compromising treatment adherence [10]. These limitations have driven the development of alternative formulations, such as glycerol phenylbutyrate (GPB), a triglyceride in which glycerol is esterified with three molecules of 4PB, which is also clinically approved (Ravicti™) for the treatment of patients with UCD [11]. This formulation retains the efficacy of 4PB in controlling pruritus and cholestasis while offering improved oral tolerability and better adherence of patients with PFIC2 [12]. We recently confirmed that GPB significantly corrects the trafficking and activity of an ER-retained (also categorised as class II) ABCB11 variant in vitro [13]. Concerning ABCB4, only one article has demonstrated a limited correcting effect of 4PB on two class II ABCB4 variants (G228R, D459H) in vitro [14]. In this context, we evaluated here the effects of 4PB and GPB treatments on three class II ABCB4 variants, I490T, I541F and L556R, already described in the frame of our previous studies [15].

## Materials and methods

### Plasmids, cell culture, transfection, and treatments

The plasmids encoding WT and mutated ABCB4 (I490T, I541F and L556R) have been described [15]. Human embryonic kidney cells (HEK-293, hereafter referred to as HEK; ATCC^®^ CRL-1573™) and human hepatocellular carcinoma HepG2/C3A cells (a derivative of HepG2, hereafter referred to as HepG2; ATCC^®^ CRL-3581™) were obtained from the American Type Culture Collection (ATCC, Manassas, VA, USA) and cultured as described [15]. Transient transfections were performed as previously reported [15], using FuGENE^®^ HD (Promega, Charbonnières-les-Bains, France) for HEK cells and JetPrime^®^ (Polyplus Transfection, Illkirch, France) for HepG2 cells. 4PB (Merck/Sigma-Aldrich, St Quentin Fallavier, France) was dissolved in PBS (1 M stock), whereas GPB (MedChemExpress, Clinisciences, Montrouge, France) was dissolved in DMSO (1 M stock). Compounds were used at final concentrations of 5 mM for 4PB and 2.5 mM for GPB, with vehicles at the same dilutions as controls. Twenty-four hours after transfection, cells were treated with the chemical chaperones for 16 h prior to further analyses.

### Indirect immunofluorescence

Indirect immunofluorescence was performed as reported [15]. In brief, HepG2 cells grown on glass coverslips were fixed and permeabilised using ice-cold methanol after transfection and drug treatment and processed using the primary antibodies (anti-ABCB4, clone P3II-26; anti-ABCC2, clone M2I-4; Enzo Life Sciences, Villeurbanne, France) and AlexaFluor^TM^-conjugated isotype-specific secondary antibodies (Thermo Fisher Scientific, Villebon-sur-Yvette, France). Nuclei were stained using Hoechst 33342 (Thermo Fisher Scientific). Images were acquired by confocal microscopy and quantification was performed: among polarised (*i.e.,* forming canaliculi with positive ABCC2 staining) ABCB4-positive cells, the percentage of cells displaying ABCB4 staining (red channel) at the canalicular membrane (*i.e.,* colocalisation with ABCC2 – green channel) was determined.

### Functional assays

ABCB4-mediated PC secretion was quantified using a fluoro-enzymatic assay, as described [16]. In brief, culture media from transfected and drug-treated HEK cells were collected after the 16-hour treatment, and total lipids were extracted, evaporated, and resuspended in PBS containing 0.1% Triton X-100. Substrate fluorescence was measured using a multiplate fluorimeter (Varioskan LUX multimode reader, Thermo Fisher Scientific) at λ_exc_: 320 nm and λ_em_: 404 nm. All measurements were performed in triplicate, normalised to ABCB4 expression levels after background subtraction, and expressed as a percentage of the PC secretion activity measured for ABCB4-WT.

### Statistical analyses

Graphics and statistical analyses (one-way ANOVA tests) were performed using Prism version 9.00 (GraphPad software). Data are expressed as means ± standard deviation (SD). A *P* value < 0.05 was considered significant with *P < 0.05; **P < 0.01; ***P < 0.001, and ns: not significant.

## Results

### GPB improves canalicular localisation of class II ABCB4 variants

**Fig. 1:**
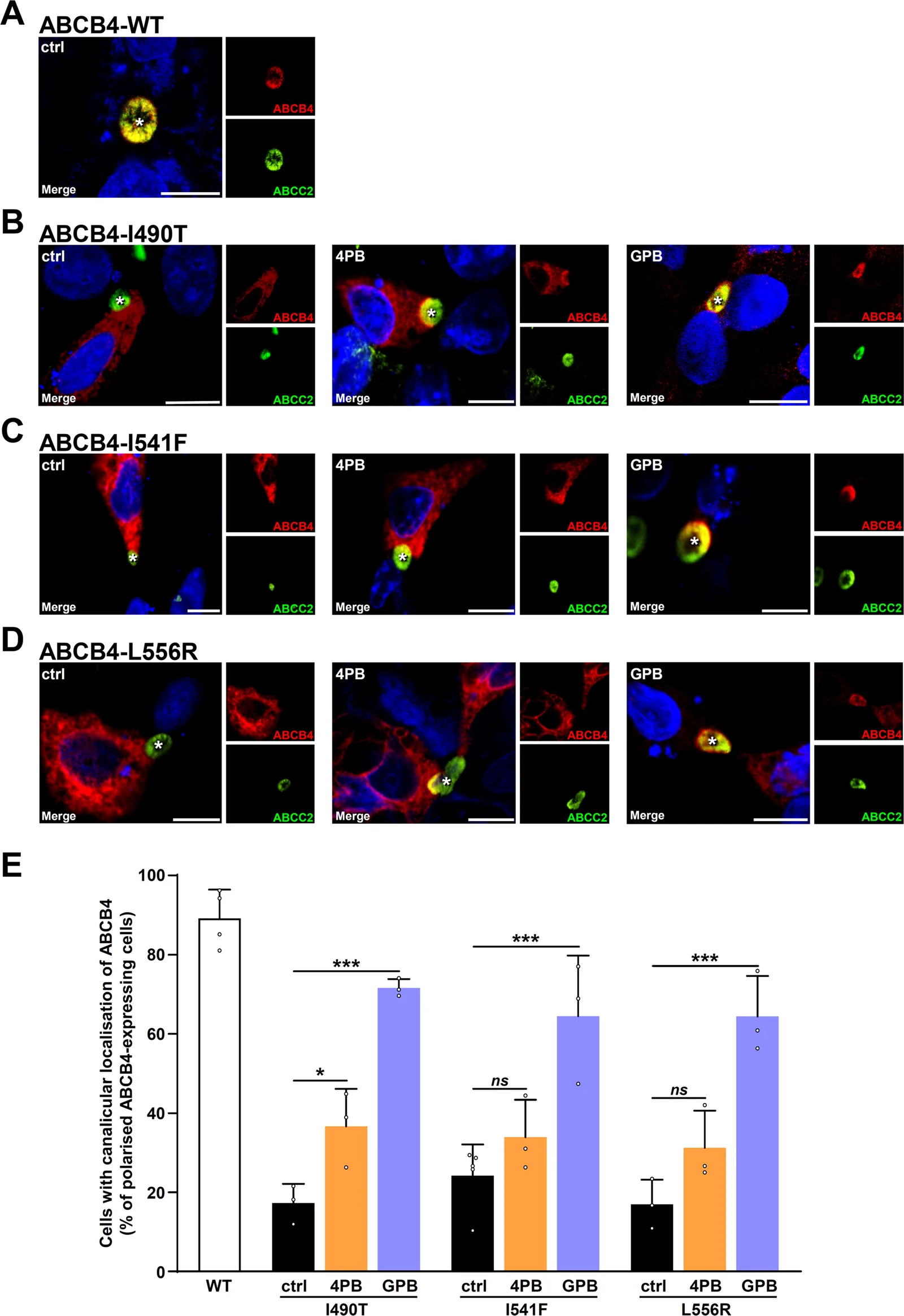
Effect of 4 PB and GPB on the canalicular localisation of class II ABCB4 variants. **A**-**D**. HepG2 cells transiently expressing ABCB4-WT (**A**), -I490T (**B**), -I541F (**C**) or -L556R (**D**) were fixed and permeabilised after 16 h of mock-treatment (ctrl), or treatment with 4 PB or GPB, as indicated. Cellular localisation of ABCB4 (red) and the canalicular marker ABCC2 (green) was then visualised by confocal microscopy after indirect immunofluorescence labelling. Nuclei are shown in the merged images (blue). These panels are representative of three independent experiments. White asterisks indicate bile canaliculi. Bars: 10 μm. **E**. Quantification of **A**-**D**. For each tested condition, the percentage of cells in which ABCB4 (WT or variant) partially or completely colocalises with the canalicular marker ABCC2 was determined by analysing > 200 cells from three independent experiments. Means (± SD) are represented

We analysed the subcellular localisation of the three class II ABCB4 variants by indirect immunofluorescence in HepG2 cells, a cell line of hepatic origin that forms pseudo-canaliculi in culture, and thus close to the pathophysiological situation. In contrast to ABCB4-WT, which predominantly colocalises with the canalicular marker ABCC2 (Fig. 1A), the three class II ABCB4 variants are retained intracellularly and do not colocalise with ABCC2 (Fig. 1B–D, left panels), as previously reported [15]. Then, we observed that both 4PB and GPB treatments increased canalicular localisation of the three ABCB4 variants (Fig. 1B-D, middle and right panels), with GPB showing greater efficiency than 4PB. Indeed, the quantification of these results indicated that GPB improved the traffic of ABCB4 variants to the canalicular membrane in 71.6 ± 2.3% (80% of ABCB4-WT), 64.4 ± 15.3% (72% of ABCB4-WT) and 64.4 ± 10.2% (72% of ABCB4-WT) of the cells for ABCB4-I490T, -I541F and L556R variants, respectively (Fig. 1E). In comparison, 4PB only allowed a canalicular localisation in 36.7 ± 9.5% (41% of ABCB4-WT), 33.9 ± 9.4% (38% of ABCB4-WT) and 31.2 ± 9.4% (35% of ABCB4-WT) of the ABCB4-positive polarised cells for ABCB4-I490T, -I541F and L556R variants, respectively, which was significant only for the I490T variant, although a similar trend was also observed for the other two variants (Fig. 1E).

### 4 PB and GPB improve PC secretion of class II ABCB4 variants

**Fig. 2:**
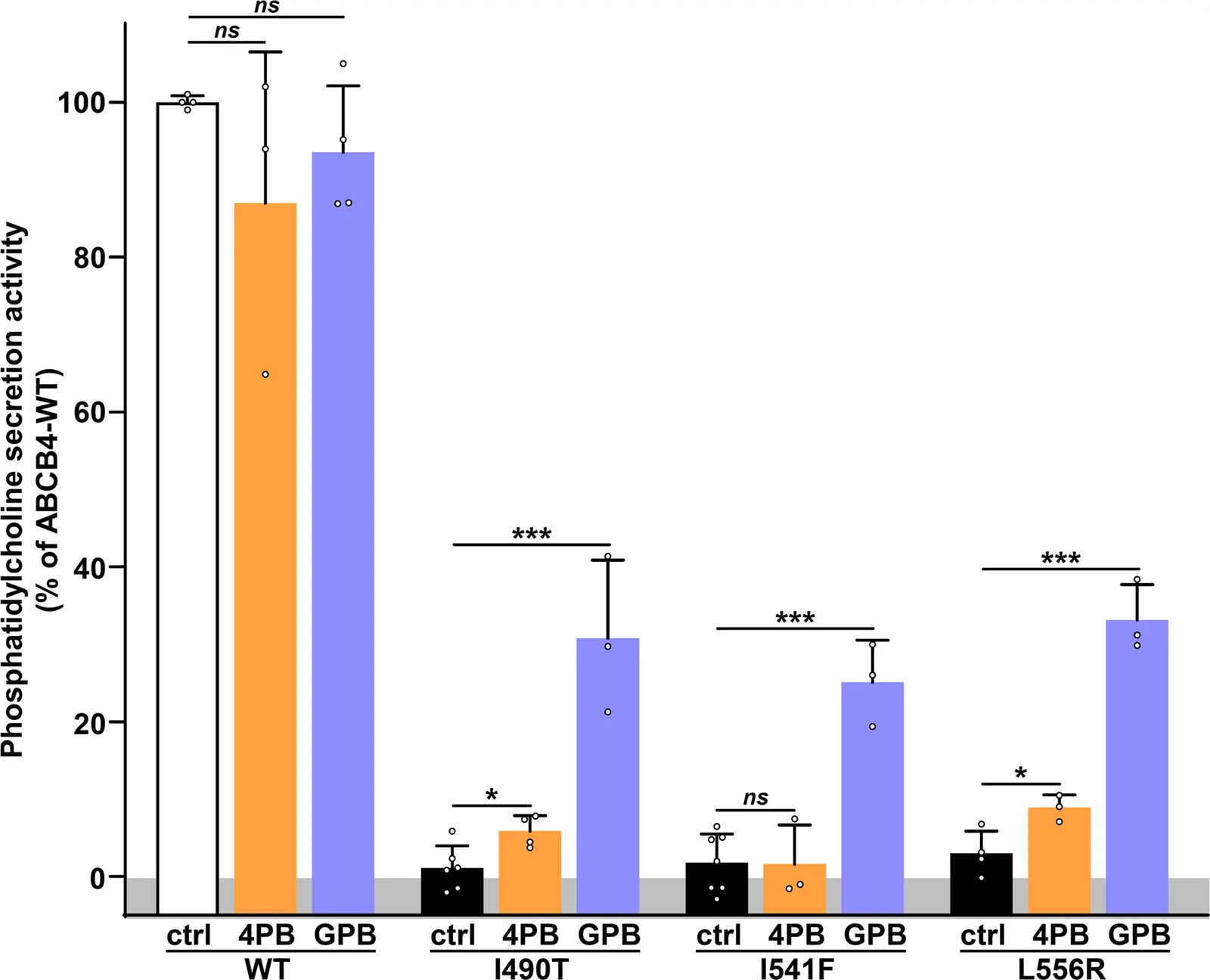
Effect of 4 PB and GPB on the PC secretory function of ABCB4-WT and class II variants. HEK cells expressing ABCB4-WT, -I490T, -I541F, or -L556R were treated with vehicle (ctrl), 4 PB, or GPB. For each condition, ABCB4-mediated PC secretion was expressed as the percentage of the activity of vehicle-treated ABCB4-WT-expressing cells. The grey area indicates values with y < 0. Means (± SD) of at least three independent experiments performed in triplicate for each condition are shown

We then performed functional analyses in HEK cells to assess the capacity of cells expressing ABCB4 variants to secrete PC after treatment with 4PB or GPB. HEK cells were used for functional assays as published [15, 16] since HepG2 cells are not suitable for such approaches due to lower transfection rates and the inaccessibility of secreted PC in the closed environment of the pseudo-canaliculi formed by the canalicular membrane of these cells. GPB was able to significantly increase the PC secretion for the three variants with 22.6 ± 8.4%, 29.9 ± 4.8%, and 33.8 ± 9.7% of ABCB4-WT activity for ABCB4-I490T, -I541F, and -L556R variants, respectively, compared to 1.1 ± 2.8%, 1.8 ± 3.7%, and 3.0 ± 2.8% of ABCB4-WT activity for ABCB4-I490T, -I541F, and -L556R variants without treatment, respectively (Fig. 2). In comparison, 4PB only increased PC secretion to 5.8 ± 2.1%, 1.6 ± 5.0% and 8.9 ± 1.7% of ABCB4-WT activity for ABCB4-I490T, -I541F, and L556R variants, respectively (Fig. 2). These results are consistent with immunofluorescence analyses, which show a greater effect of GPB than 4PB (Fig. 1E). In addition, we observed that 4PB and GPB do not inhibit ABCB4-WT function (Fig. 2) in contrast to other previously identified ABCB4 correctors [15, 16].

## Conclusion

In the context of a targeted pharmacotherapy strategy for patients with ABCB4 defects, the goal is not necessarily to obtain a total restoration of function for ABCB4 variants but rather to reach a secretion threshold that may allow a better response to UDCA treatment [5] and/or a combination with ABCC7/CFTR potentiators, e.g., VX-770/ivacaftor shown to partially rescue the function of class III ABCB4 and ABCB11 variants [17, 18]. The greater correcting potential of GPB towards 4PB may be due to the fact that GPB is composed of three phenylbutyrate moieties. Thus, 2.5 mM GPB theoretically corresponds to 7.5 mM 4PB equivalents, assuming complete hydrolysis [11]. A direct comparison with 7.5 mM 4PB was not performed because concentrations above 5 mM may exert cytostatic or cytotoxic effects in some cell models [19, 20]. In addition, the extent and kinetics of GPB hydrolysis under our cell culture conditions remain unknown, and the absence of 4PB-equivalent dose-response experiments limits direct comparison of the two compounds. Further dose-response experiments would help define the optimal concentration ranges and relative correcting efficiencies of 4PB and GPB. Glycerol has been shown to act as a chemical chaperone for class II ABCB11 variants [21] and ABCB1-I541F [22], but not strictly for ABCB4 variants. However, the maximum theoretical concentration of free glycerol that could be generated from 2.5 mM GPB upon complete hydrolysis is only 2.5 mM (approximately 0.018% v/v), which is considerably lower than the glycerol concentrations used in previous studies (7.5–10%), making unlikely a correcting effect of glycerol at such low doses. Another limitation of this study may arise from the small number of experiments carried out per condition: although three independent experiments are generally considered acceptable, this should be addressed more thoroughly in forthcoming analyses. The present in vitro study provides a proof-of-concept that GPB has therapeutic potential for selected patients with PFIC3 caused by ABCB4 missense variations affecting the trafficking of the transporter. Considering that there is an unmet medical need for patients with ABCB4 deficiency, GPB could represent a significant step forward for the care of such selected patients.

## Data Availability

The datasets used and/or analysed during the current study are available from the corresponding author on reasonable request.

## Abbreviations

4PB: 4-phenylbutyrate
ABC: ATP-binding cassette
ABCB4: ATP-binding cassette subfamily B member 4
CFTR: Cystic fibrosis transmembrane conductance regulator
ER: Endoplasmic reticulum
GPB: Glycerol phenylbutyrate
PC: Phosphatidylcholine
PFIC2/3: Progressive familial intrahepatic cholestasis types 2/3
UCD: Urea cycle disorder
UDCA: Ursodeoxycholic acid
WT: Wild type
